# Laryngeal vibration as a non-invasive neuromodulation therapy for spasmodic dysphonia

**DOI:** 10.1038/s41598-019-54396-4

**Published:** 2019-11-29

**Authors:** Sanaz Khosravani, Arash Mahnan, I-Ling Yeh, Joshua E. Aman, Peter J. Watson, Yang Zhang, George Goding, Jürgen Konczak

**Affiliations:** 1 Human Sensorimotor Control Laboratory, School of Kinesiology, University of Minnesota, Minnesota, USA; 2 Department of Occupational Therapy, Singapore Institute of Technology, Singapore, Singapore; 3 Department of Neurology, University of Minnesota, Minnesota, USA; 4 Department of Speech, Language, and Hearing Sciences, University of Minnesota, Minnesota, USA; 5 Department of Otolaryngology, University of Minnesota, Minnesota, USA

**Keywords:** Dystonia, Motor cortex, Neurophysiology

## Abstract

Spasmodic dysphonia (SD) is an incurable focal dystonia of the larynx that impairs speech and communication. Vibro-tactile stimulation (VTS) alters afferent proprioceptive input to sensorimotor cortex that controls speech. This proof-of-concept study examined the effect of laryngeal VTS on speech quality and cortical activity in 13 SD participants who vocalized the vowel /a/ while receiving VTS for 29 minutes. In response to VTS, 9 participants (69%) exhibited a reduction of voice breaks and/or a meaningful increase in *smoothed cepstral peak prominence*, an acoustic measure of voice/speech quality. Symptom improvements persisted for 20 minutes past VTS. Application of VTS induced a significant suppression of theta band power over the left somatosensory-motor cortex and a significant rise of gamma rhythm over right somatosensory-motor cortex. Such suppression of theta oscillations is observed in patients with cervical dystonia who apply effective sensory tricks, suggesting that VTS in SD may activate a similar neurophysiological mechanism. Results of this feasibility study indicate that laryngeal VTS modulates neuronal synchronization over sensorimotor cortex, which can induce short-term improvements in voice quality. The effects of long-term VTS and its optimal dosage for treating voice symptoms in SD are still unknown and require further systematic study.

## Introduction

Spasmodic dysphonia (SD) is a speech-specific focal dystonia of the larynx that typically develops in middle adulthood between 40–50 years of age^1^. SD leads to the formation of voice breaks and/or a strained or choked speech^2^. Two major classes of SD have been identified: adductor (ADSD), characterized by involuntary vocal fold closure; and abductor (ABSD), exhibiting excessive vocal fold opening^1^. Currently, SD is primarily treated with Botulinum neurotoxin injection (BoNT), which despite its effectiveness, is an invasive method and only temporarily relieves voice symptoms^3^.

The underlying neural mechanism of SD is not entirely understood, but it is known to involve structural and functional alterations in the basal ganglia–thalamo-cortical circuitry, the brainstem, and the cerebellum^4–8^. Several forms of focal dystonia, including cervical dystonia (CD), blepharospasm, and spasmodic dysphonia present with somatosensory system abnormalities even in non-dystonic muscles^9–12^. This suggests that while the motor symptoms of dystonia are focal, the corresponding somatosensory impairments are general.

Furthermore, it is known that in cervical dystonia successful sensory tricks (*geste antagoniste*) can ease dystonic symptoms by touching areas over or near the dystonic musculature; a phenomenon that sheds light on the link between abnormal somatosensation and the dystonic motor manifestations^13,14^. In addition, vibro-tactile stimulation (VTS) has been shown to reduce the severity of dystonic postures. For example, vibration over dystonic neck muscles induced immediate head righting and temporarily restored upright head posture in people with torticollis^15^. It has long been established that VTS in the range of 40–100 Hz stimulates the mechanoreceptors and muscle spindles that affect motor behavior^16–19^. However, the knowledge on the distribution and function of somatosensory receptors within the laryngeal musculature is still inconclusive. A series of neuroanatomical, histochemical, and electron microscopic studies supported the existence of muscle spindles in the larynx^20–24^, while others failed to provide support for the existence of intrafusal muscle fibers in cricothyroid and thyroarytenoid muscles^25^ or could not elicit stretch responses in these areas^26^.

Moreover, the mucosa of the epiglottis is known to have an array of mechanoreceptors responsive to mechanical stimulation between 10–70 Hz with a depression amplitude of <100μm^27,28^. It was further demonstrated that the sensory basis for the laryngeal adductor response is dependent on the stimulation of mechanoreceptors in the laryngeal mucosa in the cat and humans^26,29^. Moreover, the discovery of Krause type sensory corpuscles at the free edge of vocal cords is consistent with the notion that signals from these mechanoreceptors are not only used for the control of swallowing but also for voice control^28^.

Recent work from our group demonstrated upper limb proprioceptive deficits in SD^12^. Moreover, studies on the effects of sensory stimulation of the larynx have indicated a reduced inhibition of laryngeal muscle responses to sensory stimulation, i.e., a reduced suppression of motor responses to laryngeal sensory stimulation^5^. Functional neuroimaging in SD during phonation has also shown increased central activation in the laryngeal somatosensory cortex in patients with SD^8^. These studies highlight different forms of somatosensory abnormalities in SD, which may contribute to the pathomechanism of the disease. This notion opens an avenue for a potential behavioral treatment for SD that seeks to modulate the somatosensory information of the laryngeal musculature to improve the speech motor output. In particular, the vibro-tactile stimulation of laryngeal muscles might be a suitable tool for this purpose, given that it is shown to alter the afferent proprioceptive signals produced by the vibrated muscle mechanoreceptors and muscle spindles^16,17^.

This study sought to establish the feasibility of laryngeal VTS as a non-invasive neuromodulation treatment for spasmodic dysphonia. We pursued two specific aims: First, to demonstrate that prolonged VTS can induce short-term acute changes in speech quality. Second, to document the changes in cortical activity in SD that are associated with the application of laryngeal VTS. To evaluate the effectiveness of laryngeal VTS we had people with SD vocalize the vowel /a/ while receiving VTS for a total of 29.4 minutes. We derived markers of speech quality and analyzed the neuronal response to VTS over sensorimotor cortex.

## Results

### Improvement of measures of speech quality in response to laryngeal VTS

**Figure 1:**
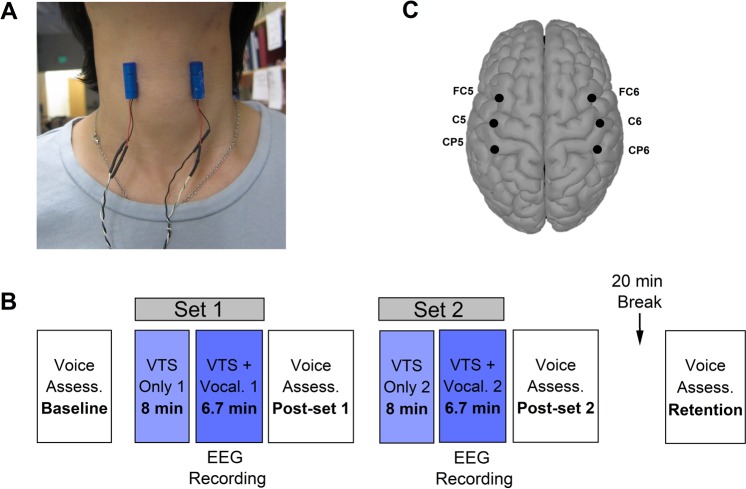
Setup for the application of laryngeal VTS and the experimental protocol. (**A**) The encapsulated vibration motors attached to the laryngeal area (lateral parts thyroid cartilage). (**B**) The experimental protocol comprised two identical sets: (1) application of laryngeal vibration for 7 minutes (*VTS Only*), and (2) vocalization and laryngeal vibration for 10 minutes (*VTS* + *Vocal*.). Total exposure to VTS was 29.4 minutes. The standard evaluation of speech quality was performed at four different stages throughout the experiment: Pretest, at the end of the first block (Post-set1), at the end of the second block (Post-set2), and 20 minutes after VTS had stopped (Retention). EEG recording occurred during the VTS + Vocalization condition.

We recorded the voice of 13 SD participants as they read a list of sentences devised for the speech evaluation of SD^30^ at 4 different time stamps along the experimental protocol: Prior to VTS (Pretest), after 14.7 minutes of VTS (Post-set 1), after 29.4 minutes of VTS (Post-set 2) and 20 minutes past the cessation of VTS (Retention) (see Fig. 1B for an overview). Subsequently, we derived the number of voice breaks and *smoothed cepstral peak prominence* (CPPS) as measures of speech quality from the acoustic signal. CPPS is based on the acoustic signal’s power spectrum and correlates strongly with the severity of SD voice symptoms^31^ (for details, see Method: Measures of speech quality).

**Figure 2:**
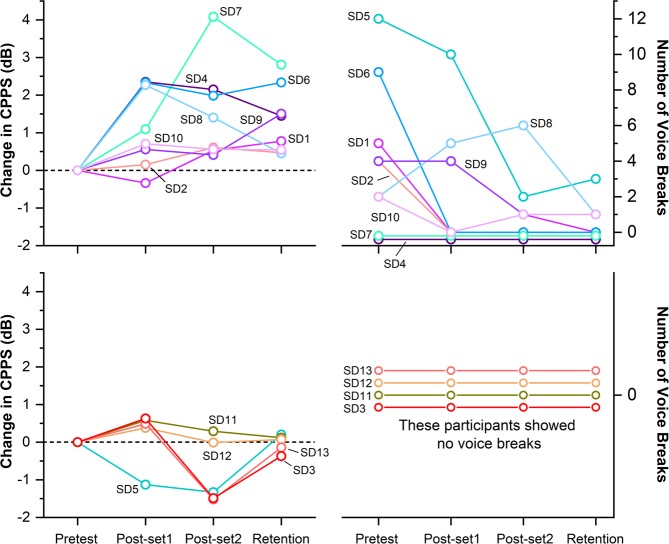
Change in the number of voice breaks and CPPS at different stages of VTS application (Pretest, Post-set1, Post-set2, and Retention). Responders to VTS are shown in the top panels, non-responders in the bottom panels). Note that all non-responders exhibited no voice breaks prior and during VTS. Typically, responders showed improvements in both markers of voice/speech quality. Note that SD 5 showed no effect in CPPS, but drastically reduced the number of voice breaks with VTS application.

Nine out of 13 participants (69%) responded to VTS and showed a reduction of the number of voice breaks and/or a rise of CPPS (>+1 dB) at Post-set 1 and/or the Post-set 2 as compared to Pretest. The remaining four participants did not show a consistent response to VTS as quantified by a rise in CPPS. It is noteworthy that none of the non-responding patients exhibited voice breaks at Pretest (see Fig. 2; bottom panels). Improvements in both speech quality measures for the responders were preserved at the retention stage (see Fig. 2; top panels). As a group, participants showed a significant rise of CPPS after 14.7 minutes of laryngeal VTS in comparison to their Pretest (*p* = *0*.*02*, *d* = *1*.*06*), which was retained at 20 minutes past the last application of VTS (*p* = *0*.*006*, *d* = *1*.*15;* see Fig. 2). The corresponding effect sizes were large (Cohen’s d >0.8) at both time stamps. In addition, 6 out of 7 SD participants (86%) who exhibited voice breaks at Pretest, showed a reduction of voice breaks in response to laryngeal VTS with 4 patients having no voice breaks after 14.7 minutes of VTS (see Fig. 2 and Table 1).

**Table 1:** VTS induced change in smoothed cepstral peak prominence (ΔCPPS) and the number of voice breaks (ΔVB) relative to baseline (Pretest). Unit for ΔCPPS is dB.

Subject ID	Post-Set 1 (ΔCPPS, ΔVB)	Post-Set 2 (ΔCPPS, ΔVB)	Retention (ΔCPPS, ΔVB)
SD1	(−0.34, −5)	(0.53, −4)	(0.78, −5)
SD2	(0.15, −4)	(0.60, −4)	(0.47, −4)
SD3	(0.63, 0)	(−1.50, 0)	(−0.37, 0)
SD4	(2.35, 0)	(2.15, 0)	(1.45, 0)
SD5	(−1.13, −2)	(−1.33, −10)	(0.2, −9)
SD6	(2.33, −9)	(1.98, −9)	(2.34, −9)
SD7	(1.10, 0)	(4.08, 0)	(2.81, 0)
SD8	(2.27, +3)	(1.40, +4)	(0.45, −1)
SD9	(0.56, 0)	(0.41, −3)	(1.50, −)
SD10	(0.71, −2)	(0.56, −1)	(0.55, −1)
SD11	(0.58, 0)	(0.29, 0)	(0.12, 0)
SD12	(0.38, 0)	(−0.01, 0)	(0.07, 0)
SD13	(0.49, 0)	(−1.52, 0)	(−0.13, 0)

### Change in the cortical oscillatory behavior in response to laryngeal VTS

**Figure 3:**
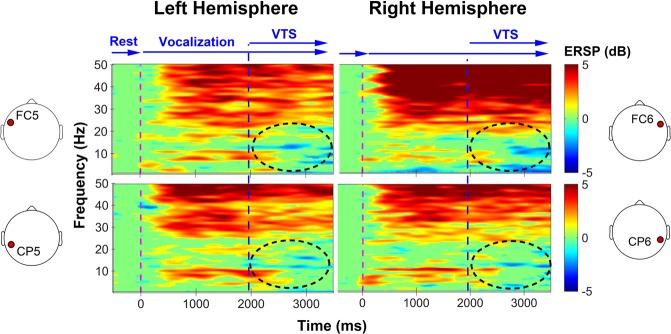
Effect of laryngeal VTS on bilateral right somatosensory and premotor cortical ERSP for VTS-off (0–2000 ms) versus VTS-on (2000–4000 ms) in a single patient. Note that laryngeal VTS resulted in the event-related desynchronization of low-frequency oscillations over somatosensory-motor cortical areas in both hemispheres (see dashed ellipses).

**Figure 4:**
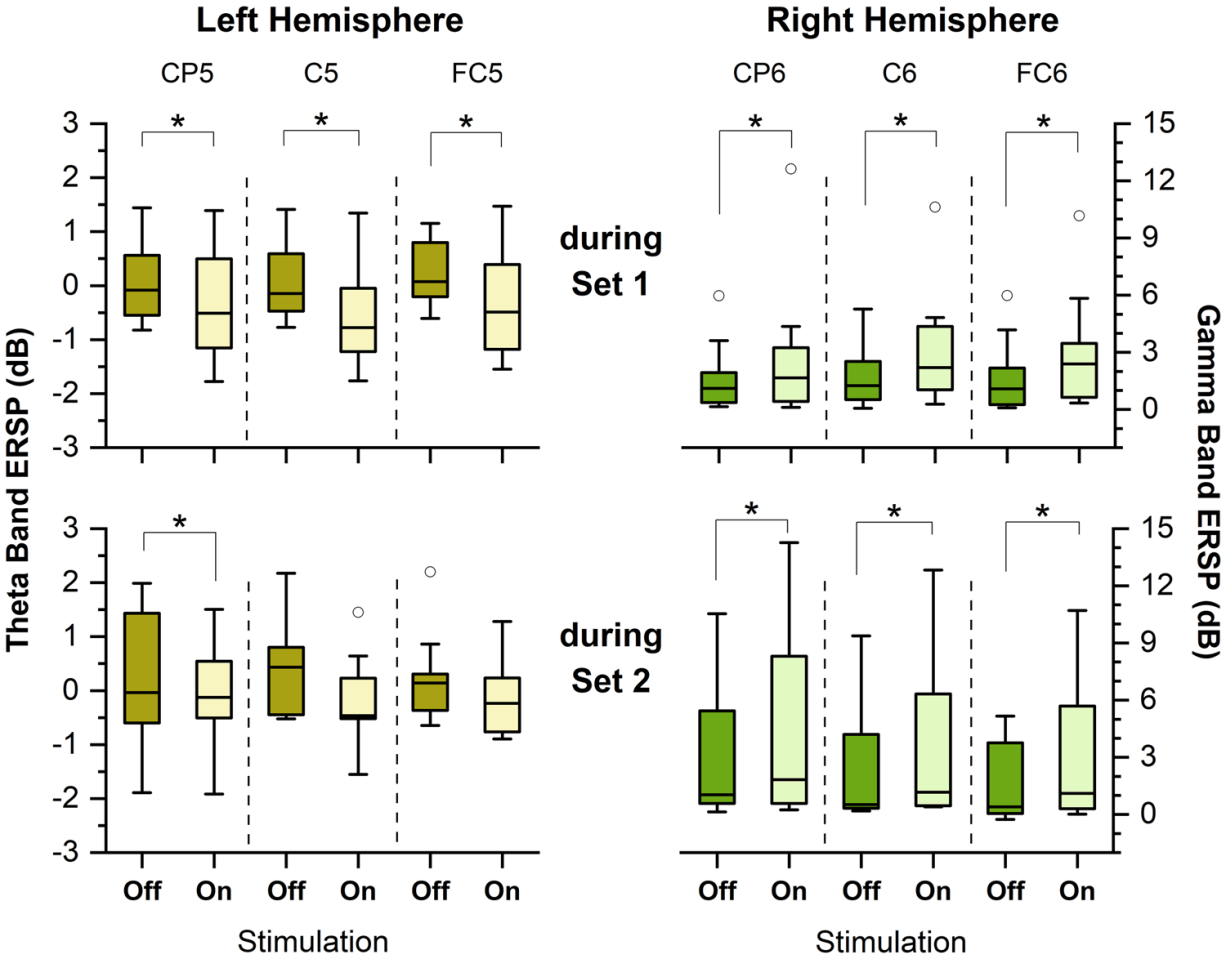
Effect of VTS on ERSP over somatosensory and motor cortical areas for theta and gamma bands during vocalization. Boxplots reflect the data for all subjects during the two sets of VTS (see Fig. 1B for the timing of the sets). Left panels: Theta band ERSP for left somatosensory (CP5), motor (C5), and premotor (FC5) cortical electrodes after the first and second set of VTS. Right panels: Gamma band ERSP over right somatosensory (CP6), motor (C6), and premotor (FC6) cortical electrodes after the first and second application of VTS. The boxplots represent the distribution of individual ERSP values within each group. The lower and upper boundaries of each box depict the 25% and 75% quartiles, respectively. The horizontal line within the box indicates the median. The upper and lower whiskers extend to +1.5 and −1.5 inter-quartile range, respectively. Outliers are shown as white circles. *Indicates a p-value of <0.05.

The effect of VTS on cortical oscillatory activity over somatosensory and premotor cortex resulted in an almost immediate suppression of low-frequency oscillations as illustrated in an exemplar time-frequency plot of one SD participant in Fig. 3. For the complete patient sample, the application of VTS during the first 14.7 minutes (Set 1; see Fig. 1B) induced a significant event-related desynchronization of cortical theta-band oscillations over the left somatosensory, motor, and premotor cortex (C5: *p* = *0*.*049*, *d* = *0*.*82*; CP5: *p* = *0*.*049*, *d* = *0*.*47*; FC5: *p* = *0*.*049*, *d* = *0*.*65;* see Fig. 4, top panels), and a significant immediate rise of the somatosensory and motor cortical gamma power over the right hemisphere: (*C6: p* = *0*.*037*, *d* = *0*.*48*; CP6: p = *0*.*037*, *d* = *0*.*39*; FC6: *p* = *0*.*029*, *d* = *0*.*51*). After participants had received VTS in Set 2, a similar pattern of cortical activity emerged. It again resulted in a significant desynchronization of theta oscillations over the left motor cortical area (C5: *p* = *0*.*012*, *d* = *0*.*83*), and a significant rise of gamma oscillations over the right somatosensory-motor cortical regions: (*C6: p* = *0*.*015*, *d* = *0*.*38*; CP6: p = *0*.*027*, *d* = *0*.*36*; FC6: *p* = *0*.*015*, *d* = *0*.*47*; see Fig. 4, bottom panels).

There were no significant changes of theta spectral power over the right hemisphere, or of gamma band power over the left hemisphere. Similarly, assessment of ERSP in other frequency bands (alpha and beta) did not reveal any significant changes pre- versus post-VTS for any of the electrodes over left/right hemispheres (all *p’s* > *0*.*05*).

We also performed a Pearson’s correlation analysis between the change in behavioral markers of voice quality (CPPS or the number of voice breaks) and theta/gamma ERSP for all participants collectively (responders and non-responders). No significant correlational relationships were observed. We then repeated the same analysis only on the responder group for whom either a rise in the CPPS or a decline in the number of voice breaks was observed (SD1, SD2, SD4, SD5, SD6, SD7, SD8, SD9, SD10). Again, no significant relationships were found.

A subsequent coherence analysis examined potential differences in the spectral characteristics of somatosensory-motor cortical interactions in each hemisphere. This analysis found no evidence that laryngeal VTS significantly affected the inter-regional spectral coherence between somatosensory and motor cortical areas within each hemisphere (all *p’s* > *0*.*05*).

## Discussion

This pilot-feasibility study explored whether laryngeal vibro-tactile stimulation can provide benefits for patients with spasmodic dysphonia by monitoring its short-term effects on voice quality and the associated activity over laryngeal somatosensory-motor cortical areas. The main findings of this research are as follows: First, a one-time application of laryngeal VTS resulted in the significant improvement of two standard measures of voice/speech quality in 69% of the patients. The effect persisted for at least 20 minutes after the cessation of VTS. What seemed to discriminate the “responders” from those participants, who received little to no benefits from VTS, was that “non-responders” were more mildly affected and had no voice breaks prior to receiving VTS (see Fig. 2). Second, the application of laryngeal VTS induced an immediate significant suppression of theta band synchronization over the left somatosensory-motor cortex and the immediate significant rise of gamma band synchronization over the right somatosensory-motor cortical region.

### Possible mechanisms behind the effectiveness of laryngeal VTS for improving speech in SD

Abnormal kinaesthetic function has been reported in non-dystonic limbs and muscle systems in SD^12^ and other forms of focal dystonia such as blepharospasm and cervical dystonia^10^. This implies that a more generalized somatosensory deficit underlies or is associated with the focal motor dysfunction in dystonia. We here explored if modulating somatosensory inputs could provide an avenue for a missing behavioral treatment for SD. Our approach of applying vibro-tactile stimulation constitutes a form of non-invasive neuromodulation that alters the output of afferent proprioceptive and tactile mechanoreceptors^16,17^, which is then centrally processed. Among the prominent neuropathological features of dystonia are reduced neuronal discharge rates and altered discharge patterns within the basal ganglia-thalamo-cortical motor circuitry^32^. Invasive neuromodulation techniques, such as deep brain stimulation, attempt to normalize the irregular neuronal discharge patterns by applying high-frequency impulses to targeted subcortical nuclei^33,34^ with the aim to restore the activity of upstream motor cortical networks. Here we suggest that a non-invasive high-frequency peripheral stimulation via laryngeal VTS may similarly modulate the discharge patterns of neurons in the somatosensory-motor speech network^35^, which can positively affect the speech motor output in SD.

### Modulation of cortical oscillations in response to laryngeal VTS

We recorded EEG signals to understand how laryngeal VTS affects cortical activity in SD. We found that in our sample of SD participants applying laryngeal VTS was associated with a significant suppression of theta band power oscillations over the left somatosensory-motor cortex and a significant rise of gamma rhythm over right somatosensory-motor cortex (see Fig. 4). Theta oscillations are detectable in a number of brain nuclei, including the *striatum*^36,37^. Previous research identified abnormal theta oscillations at subcortical and cortical levels in other forms of focal dystonia such as cervical dystonia^38,39^. These abnormal theta oscillations in *globus pallidus internus* significantly correlate with the severity of symptoms in cervical dystonia^40^.

The susceptibility of focal dystonia to somatosensory stimulation has long been recognized as patients with task-specific dystonia may use sensory tricks (*geste antagoniste*) to alleviate dystonic symptoms temporarily by touching or pressing areas of or near the dystonic musculature. The neurophysiological correlate of an effective sensory trick is the suppression of abnormal cortical theta oscillations in CD^41^. The similarity between our EEG finding of suppressed theta band power in SD and the one reported for patients with CD^41^, suggests that the improvement of abnormal speech motor output in SD via laryngeal VTS may activate the same neurophysiological mechanism underlying an effective sensory trick in CD.

Another identified feature of modulated sensorimotor cortical processing due to VTS was the rise of gamma rhythm over right somatosensory-motor cortex. Gamma band oscillations are believed to form through the activation of excitatory pyramidal neurons and inhibitory interneurons regulated by the GABA-mediated synaptic current^42^. The synchronization of gamma oscillations underlies task-specific functions such as somatosensory processing^43^ and motor preparation^42,44^. Gamma activity in the 40 Hz range has been detected during speech^45^. Movement-induced changes in gamma amplitude seem to reflect the processing of afferent proprioceptive feedback in motor cortex^46,47^. Moreover, a rise of subcortical gamma-band synchronization correlates with the amplitude and velocity of hand movements, highlighting its involvement in the neural control of movement^48^. Given the empirical evidence showing that cortical gamma band activity underlies volitional motor control, our finding of a VTS-induced rise of gamma oscillations cortical areas involved in voice and speech motor control, indicates that laryngeal VTS alters information processing within speech cortical networks, which positively influences the voice quality of people with SD.

### Limitations of the study

This proof-of-concept study yielded initial evidence that laryngeal VTS can improve voice symptoms in SD. A main limitation of this study is the lack of a control SD group that would allow for the systematic examination of possible confounding placebo or practice effects. Although we cannot exclude the possibility that the observed improvements in voice symptoms constitute a placebo effect, we do know from our pilot work that attaching the vibrators to the skin above the voice box (without being turned on) does not improve voice quality in SD. That is, it is unlikely that mere tactile stimulation would suffice in reducing voice symptoms. Moreover, there are no reports indicating that touching the neck constitutes a widely used and effective sensory trick in SD. In addition, the observed improvements in voice quality are not explained as a Hawthorne or special attention effect. On the contrary, as these patients were tested in their symptomatic stage when speech production is exhausting and effortful, one would expect that repeated vocalization and speech over more than 30 minutes results in a decline of speech, which we did not observe. Participants had not practiced the relevant test sentences prior to testing, nor is there evidence that voice symptoms in SD subside with repeated and prolonged speech. Finally, the effects on speech were observed when VTS was not applied. We recorded speech always after the end of each set of VTS (see Fig. 1B). In addition, the positive effects on markers of speech lasted for 20 minutes after the cessation of VTS.

A different drawback concerns the lack of an objective established clinical scale to classify disease severity. Understanding why and how disease severity interacts with laryngeal VTS could be very useful in predicting who would respond well and would likely be a non-responder to VTS. We choose CPPS and the number of voice breaks as prominent predictors of SD severity^49^. The inclusion of other outcome measures such as the consensus auditory-perceptual evaluation of voice (CAPE-V)^50^ may provide additional markers for examining the effectiveness of laryngeal VTS. In summary, obtaining additional outcome measures to characterize disease severity in SD and then testing the effects of VTS in a larger sample of SD patients would be clinically meaningful in understanding who responds well to laryngeal VTS and who will likely not benefit from this treatment.

## Conclusions

This is the first study that investigated the effect of laryngeal VTS on SD voice symptoms. Its results lay the scientific foundation for a randomized clinical trial to examine the usefulness of the approach in a larger patient sample and to document the longitudinal changes in voice quality and the underlying cortical responses to laryngeal VTS in SD. Such clinical trial must address the shortcomings of this feasibility study. In a first step towards translating this knowledge into a clinical application, we are currently conducting a clinical trial, in which people with adductor SD undergo an 8-week training, in which they apply laryngeal VTS in-home (ClinicalTrials.gov Identifier: NCT03746509). Its results should solidify our knowledge on the effectiveness of VTS for treating the voice symptoms in SD.

The current study showed that the application of laryngeal VTS can result in meaningful improvements of speech quality in SD. Laryngeal VTS induced a significant suppression of theta band power over the left somatosensory-motor. A similar suppression of theta oscillations is observable in cervical dystonia patients applying effective sensory tricks, suggesting that VTS in SD may activate a similar neurophysiological mechanism.

## Methods

### Participants

Thirteen people with SD (8 female, 5 male; mean age ± SD: 58.6 ± 12.5 years) were recruited through the University of Minnesota Fairview Lion’s Voice Clinic. Patients receiving Botulinum neurotoxin were tested toward the end of their injection cycle when they are most symptomatic (see Tables 2 and 3 for clinical characteristics). This study was approved by Institutional Review Board of the University of Minnesota. All study participants gave written informed consent prior to study begin. No human participants under the age of 18 years were recruited for this study. The experiment was conducted in line with the relevant guidelines and regulations. The clinical trial related to this work is registered with clinicaltrials.gov (*Study identifier*: *NCT03746509*; first posted on 19 November 2018*)*.

**Table 2:** Clinical characteristics of study participants.

Subject ID	Gender	Age	Dominant Hand	SD type	Diagnosis Duration (mo.)	BoNT cycle (mo.)	Last BoNT Injection (mo.)
SD 01	Female	71	Right	Adductor	44	4	9
SD 02	Female	48	Right	Adductor	48	4	3
SD 03	Male	59	Right	Adductor	40	4.5	4.5
SD 04	Male	60	Right	Adductor	50	>3	3
SD 05	Male	73	Right	Abductor	180	NA	36
SD 06	Female	57	Right	Adductor	36	NA	NA
SD 07	Female	62	Right	Adductor	411	5	2
SD 08	Male	26	Right	Adductor	93	2–5	2.5
SD 09	Female	65	Right	Adductor	204	2	2
SD 10	Female	56	Right	Adductor	324	6	6
SD11	Female	57	Right	Adductor	15	3	3
SD12	Male	74	Right	Adductor	396	4	4
SD13	Female	54	Right	Adductor	288	3	3.5

**Table 3:** Clinical and self-perceived markers of speech and voice symptom severity for study participants.

Subject ID	Number of Voice Breaks	Voice Tremor	Self-Rated Effort Scale (Adductor/Abductor Sentences)	Self-Rated Effort Scale (Vowel /a/)
SD 01	5	Moderate	4	7
SD 02	4	No	3	2
SD 03	0	No	2	3
SD 04	0	No	2	3
SD 05	12	No	3	2
SD 06	9	Moderate	5	7
SD 07	0	Mild to moderate	4	5
SD 08	2	No	7	6
SD 09	4	Mild to moderate	9	8
SD 10	2	Mild	2	2
SD 11	0	No	2	3
SD 12	0	No	2	2
SD 13	0	No	3	3

A potential concern when examining SD patients medicated with BoNT is vocal fold immobility, often occurring at higher dosage, one-sided BoNT injections (>5 units). Nevertheless, in this study, patients were given bilateral low-dose injections (0.2–2 units per vocal fold), while the dosage of injection was determined according to the severity of voice symptoms. This technique reduces the possibility of occurrence of vocal fold immobility. Another concern might be the bowing of vocal folds that occasionally appears shortly after BoNT injections. However, this condition also disappears with the improvement of voice/re-emergence of vocal spasms^51^. Accordingly, because the experimental session was held only after the recurrence of the symptoms, it was unlikely that vocal fold immobility/bowing occurred in our sample of study participants.

### Apparatus

As stimulators, we used a pair of lightweight encapsulated vibro-motors (Model 307–100, Pico Vibe, Precision Microdrives Ltd., London, UK; diameter: 8.8. mm, length 25 mm). The vibro-motors were attached bilaterally on the lateral area of the thyroid cartilage at the height of vocal folds. Preliminary work in our laboratory with healthy human volunteers showed that a vibration frequency of 100 Hz with these vibrators generates peaks in the power spectrum of the voice signal that are within the frequency range known to stimulate laryngeal mechanoreceptors in animals^27^ or induce kinaesthetic illusions in humans which are known to be based on muscle spindle input^18,19^. Accordingly, the vibration frequency for VTS was set at100Hz in this study. Thus, we could reasonably assume that besides the tactile receptors of the skin above the voice box, laryngeal mechanoreceptors were also stimulated. At 100Hz, vibration frequency the vibration amplitude of the vibro-motors was ~1.7 G (1 G = 9.81 m/s^2^).

Electroencephalographic (EEG) data were recorded with the ActiveTwo data acquisition system (Biosemi B.V. Ltd, Amsterdam, Netherlands). The sampling rate was set at 512 Hz. Brain potentials were captured via Biosemi’s 64-channel EEG cap with an equiradial system of electrode placement. A series of 250 ms long auditory cues (1000 Hz, 98 dB) generated by RPvdsEx software (Tucker-Davis Technologies Ltd., Alachua, USA) guided the study participants throughout the experiment. The auditory stimuli were presented via a pair of sound delivery tubes embedded in the left and right ear canals. The tubes were surrounded by disposable foam earplugs, which masked any auditory inputs to the ears except for the presented auditory stimuli. The same system was used to control the activation of the vibro-motors. The time-stamp of auditory cues and vibration onset/endpoint were captured simultaneously.

### Experimental procedure

The experiment took place in a chamber that was electrically and acoustically isolated. Participants were seated on a comfortable chair, asked to avoid extra movements, and to focus their attention at a fixed point on the front wall. A pair of vibro-motors was attached to the skin over the participant’s laryngeal area (see Fig. 1A). Prior to the experiment, the severity of speech symptoms was evaluated by (1) reading aloud a series of standard SD symptom-eliciting sentences^30^; and (2) pronouncing the vowel /a/ three times, each lasting four seconds. Participants pronounced the vowels and read the sentences at their habitual pitch and loudness. All speech and voice signals were recorded for later offline analysis.

The experimental protocol comprised two blocks: (1) laryngeal vibration (VTS Only), and (2) vowel vocalization accompanied by laryngeal VTS (Vocalization + VTS). During the VTS Only condition, the laryngeal vibrators were alternately turned on and off (3 seconds ON following 3 seconds OFF), for 50 repetitions and then stayed ON continuously for the final 3 minutes. During the Vocalization + VTS condition, participants received an auditory cue (1000 Hz, 98 dB) for 250 ms, and then vocalized the vowel /a/ continuously for 4 seconds. During the second half of this vocalization period, laryngeal VTS was applied (see Fig. 3). Participants stopped vocalization with the cessation of laryngeal VTS. This procedure was repeated 50 times with 4-second long resting intervals in between trials. Participants received VTS in two sets with each set lasting 14.7 minutes. Between sets, at the end of set 2, and 20 minutes after the cessation of VTS (Retention), we evaluated voice/speech quality using the same assessment tasks given at Pretest (see Fig. 1B). The duration of the retention period was arbitrarily picked between the minimum and maximum duration of VTS application (>14.7 min and <29.4 min). For further details, see S2. Supplementary Notes.

### Measures of speech quality

Participants read two sets of standard sentences^30^ devised for the speech evaluation of people with adductor and abductor spasmodic dysphonia in their normal conversational style (see S1 Supplementary Methods). Assessment of these recorded voice data was performed offline. Two voice measures were obtained: (1) the number of voice breaks (VB), and (2) the change in the cepstral peak prominence (CPP) of voice^52^. CPP is an acoustic measure of speech quality defined as the logarithm of the Fourier Transform of the signal’s power spectrum. CPP is the difference between the amplitude of the cepstral peak and the estimated value on the regression line right below the cepstral peak. The higher the relative amplitude of the cepstral peak of a voice signal, the more a well-defined harmonic structure of the voice exists. Subsequently, the CPP signal was smoothed by averaging the cepstral magnitude across frequencies and time^31^. The smoothed measure of CPP referred to as the CPPS is strongly correlated with the overall dysphonia severity^53^. In our analyses, speech signals were broken into ‘voiced’ and ‘voiceless’ segments and CPPS values were derived only for the ‘voiced’ periods.

The PRAAT software^54^ was implemented for the acoustic analysis of the voice data and the derivation of CPPS values. A certified speech-language pathologist identified voice breaks by analyzing the continuous speech of the spoken sentences. Voice tremor was identified in the sustained vowels by examining the pitch tracing and the upper harmonics in narrow-band spectrograms using the PRAAT.

### EEG signal processing and electrocortical measures

The EEGLab toolbox of MATLAB (The MathWorks, Natick, MA) was used for exploring the EEG data^55^. The averaged signal of the two external electrodes embedded over bilateral mastoid bones was used to reference all electrodes. The data were high-passed filtered at the cut-off frequency of 1 Hz to address possible baseline drifts. A zero-phase notch filter was used to remove power line noise. Next, in order to weaken the potential effect of non-cortical sources that might have been commonly captured by electrodes, each channels was re-referenced to the common average of all electrodes. Segments of EEG recordings from 1000 ms before vocalization to 4000 ms after the onset of vocalization were extracted as data epochs. We subsequently used the ‘runica’ algorithm to perform independent component analysis (ICA) on all data channels. This was followed by the implementation of an automated multiple artifact rejection algorithm ‘SASICA’^56^ on the resultant components to identify and remove the contaminated ICs. This algorithm recruits spatiotemporal criteria to distinguish the artifactual components. This is critically important for the identification and removal of muscle artifacts that may have contaminated the EEG data during vowel vocalization. At the end, the remaining ICs were linearly summed up and the output dataset was used for extracting the features.

As primary EEG measure, we obtained the event-related spectral perturbation (ERSP) of somatosensory-motor cortical electrodes in response to VTS. ERSP presents the logarithm of the mean event-related alteration in spectral power relative to the resting state at each frequency bin^57^. Band-specific features were extracted for the physiologically-relevant frequency ranges (i.e. <50 Hz): theta (4–8 Hz), alpha (8–13 Hz), beta (13–30 Hz), and the low gamma (30–49 Hz). ERSP was extracted for six sites: CP5, C5, FC5, CP6, C6, and FC6. CP5 and CP6 were nearby bilateral somatosensory cortical areas. C5 and C6 were nearby bilateral motor cortical areas. FC5 and FC6 were nearby bilateral premotor cortical regions. Indices ‘5’ and ‘6’ reflect the cortical areas close to bilateral vocalization regions over somatosensory and motor homunculi^58,59^.

As secondary EEG measure, we obtained the event-related coherence (ERCOH) between pairs of somatosensory-motor cortical electrodes as an indicator of the level of synchrony between the two electrodes^60^. ERCOH was derived for CP5-FC5 and CP6-FC6 electrode pairs. Before the computation of ERCOH, EEG epochs were pre-whitened to exclude possible autocorrelations/trends that might interfere with the data.

EEG features were extracted from the Vocalization + VTS conditions of both sets (see Fig. 1B) to investigate the immediate cortical response to VTS. For each condition, the 4000 ms long trials were divided into two segments: (1) VTS-off (before the onset of laryngeal vibration), and (2) VTS-on (after the onset of laryngeal vibration). For each participant, the ERSP measure of the average of the 50 recorded epochs was derived separately for the VTS-off and VTS-on segments. Because the first 500 ms of the VTS-off period additionally contain cortical auditory evoked potentials^61^ or be influenced by the reaction time of the study participants^62^, the first 500 ms of vocalization were excluded from further EEG analysis (i.e. the VTS-off interval was defined between 500–2000 ms after the presentation of the auditory cue).

### Statistical analysis

For SD1 to SD3 no EEG data were available. Statistical comparisons of the pre- versus post-VTS cortical potentials were performed on the available EEG data of 10 participants. The Kolmogorov-Smirnov test was implemented to examine the normality of the data. Since the distribution of the data was not normal, the non-parametric Wilcoxon sign rank test was used for statistical assessments. For each frequency band and for the group of electrodes covering each hemisphere, p-values were adjusted for multiple comparisons using the Benjamini-Hochberg method^63^. The significance level was set at p-value = 0.05. The effect size was calculated using Cohen’s d.

## Supplementary information

Supplementary Materials

## Data Availability

The datasets generated under this study are available from the corresponding author on a reasonable request.
