# Supplementary material for: Laryngeal vibration as a non-invasive neuromodulation therapy for spasmodic dysphonia
Source: Sci Rep. 2019 Nov 29;9:17955. doi: 10.1038/s41598-019-54396-4 (PMC6884515; doi:10.1038/s41598-019-54396-4)
Supplement: Supplementary file 1 — Supplementary Materials [file 41598_2019_54396_MOESM1_ESM.pdf]

SUPPLEMENTARY MATERIALS

Laryngeal vibration as a non-invasive neuromodulation therapy for spasmodic dysphonia

Sanaz Khosravani, Arash Mahnan, I-Ling Yeh, Joshua E. Aman, Peter J. Watson, Yang Zhang, George Goding, & Jürgen Konczak

S1. Supplementary Methods

The following sentences were used for the evaluation of speech quality in spasmodic dysphonia. The sentences are based on [1].

For the evaluation of speech quality in *adductor spasmodic dysphonia*:

1. Tom wants to be in the army.
2. We eat eels every day.
3. He was angry about it all year.
4. I hurt my arm on the iron bar.
5. Are the olives large?
6. John argued ardently about honesty.
7. We mow our lawn all year.
8. Jane got an apple for Ollie.
9. A dog dug a new bone.
10. Everyone wants to be in the army

For the evaluation of speech quality in *abductor spasmodic dysphonia*:

1. He is hiding behind the house.
2. Patty helped Katty carve the turkey
3. Harry is happy because he has a new horse.
4. During babyhood, he had only half a head of hair.
5. Who says a mahogany highboy isn't heavy?
6. Boys were singing songs outside of our house.
7. The puppy bit the tape.
8. See, there's a horse across the street.
9. Sally fell asleep in the soft chair.

10. The policy was suggested in an essay on peace.

S2. Supplementary Notes: Description of Research Protocol

Project Summary

Rationale: Spasmodic dysphonia (SD) is a task-specific focal dystonia affecting the laryngeal musculature. Voice symptoms are persistent and mainly appear during speech. SD is associated with somatosensory dysfunction. Vibro-tactile stimulation (VTS) of laryngeal muscle spindles/mechanoreceptors modulates the afferent proprioceptive signals reaching the sensorimotor cortex. Thus, VTS may constitute a form of non-invasive neuromodulation affecting somatosensory-motor cortical processing. **Objectives:** This study examined the feasibility of applying laryngeal VTS for improving speech quality in SD. **Population:** Patients with SD in their symptomatic state, i.e. not medicated or at the end of their Botulinum toxin cycle. **Methods:** SD participants vocalized the vowel /a/ while receiving VTS for a total of 34 minutes. The acoustic voice signals and EEG were recorded. Based on the acoustic signal, the number of voice breaks, and the change in the cepstral peak prominence (CPP) of voice were derived as objective markers of speech quality. Based on the EEG signals, event-related spectral perturbation (ERSP) of somatosensory and motor cortical electrodes in response to VTS was obtained. **Duration:** This was a single session feasibility study. Participants received VTS in two sets of 17 minutes each. **Design:** Single group, single treatment pre-posttest design with retention testing after 20 minutes past posttest. **Expected outcomes:** Results of this proof-of-concept study shall indicate the effect of laryngeal VTS on markers of voice quality and provide neurophysiological data on the neural correlate of VTS, i.e. how neuronal synchronization over sensorimotor cortex is affected by VTS.

General Information

Protocol title: Laryngeal Vibration in Dystonia

Name and address of the sponsor/funder: The study was funded by National Institutes of Health NIH 1R21DC011841 to Peter Watson and Jürgen Konczak, and by NIH 1 R01 DC016315-01A1 to Jürgen Konczak.

Researchers: Sanaz Khosravani, Arash Mahnan, I-Ling Yeh, Joshua E. Aman, Peter J. Watson, Yang Zhang, George Goding, & Jürgen Konczak

- SK, AM, I-LY, and JK are affiliated with Human Sensorimotor Control Laboratory, School of Kinesiology, University of Minnesota, U.S.A
- I-LY is affiliated with the Department of Occupational Therapy, Singapore Institute of Technology, Singapore
- Joshua Aman is affiliated with the Department of Neurology, University of Minnesota, U.S.A
- Peter Watson and Yang Zhang are affiliated with the Department of Speech, Language, and Hearing Sciences, University of Minnesota, U.S.A
- George Goding is affiliated with the Department of Otolaryngology, University of Minnesota, U.S.A

Address and telephone numbers of the research sites. The corresponding author and the first author are affiliated with the Human Sensorimotor Control Laboratory, School of Kinesiology, University of Minnesota, 1900 University Ave, SE, Cooke 400, 55455**.** Phone number: 612-646-4370. Testing was conducted at the Center for Applied and Translational Sensory Sciences, Elliott Hall, 75 E. River Parkway, Minneapolis, MN**.** E-mail: catss@umn.edu, Phone: 612-624-7846.

Rationale and background information. Spasmodic dysphonia (SD) is an adult-onset type of focal dystonia affecting the larynx[2]. It is task-specific and corresponds to the generation of a strained or choked speech [3]. SD has been classified in two major categories: adductor SD, typified by uncontrolled vocal fold closure; and abductor SD, characterized by uncontrolled vocal fold opening. The underlying neural mechanism of the disease is still being explored; accordingly, available treatment options for the disease are mostly symptomatic. SD is primarily treated with Botulinum toxin injection (Botox), which provides temporary symptom relief to some patients [4]. The pathophysiology of SD dominantly involves the basal ganglia–thalamo-cortical circuitry [5]. This includes atypical long-latency responses to peripheral nerve stimulation [6], reduced cortical inhibition [7], loss of axonal density and myelin content in the *genu capsula interna* where the head and neck muscles are represented [8], subtle brainstem abnormalities in the reticular formation, and mild degeneration and depigmentation of the *substantia nigra* and the *locus coeruleus* [9]. Several forms of focal dystonia, including the SD have shown somatosensory abnormalities even in non-dystonic limbs [10-13], reflecting that while the motor symptoms of the disease are focal, the corresponding somatosensory impairments are general. It is also known that in some cervical dystonia patients the application of tactile sensory tricks can alleviate dystonic symptoms [14,15]. Additionally, vibro-tactile stimulation (VTS) has been shown to reduce the severity of dystonic postures[16]. VTS in the range of 40-100 Hz can

stimulate the mechanoreceptors and muscle spindles that affect motor behavior [17-20]. Our previous work also confirmed arm proprioceptive deficits in SD [13], indicating a generic form of somatosensory abnormality which may contribute to the pathomechanism of the spasmodic dysphonia. This sets the basis for a potential behavioral treatment for SD that seeks to modulate the somatosensory afferents of the laryngeal musculature to improve the final speech motor output.

Study goals and objectives. This research evaluated the feasibility of laryngeal VTS as a non-invasive behavioral treatment for spasmodic dysphonia. We pursued two specific aims: to demonstrate that prolonged VTS can induce short-term acute changes in speech quality; and to document the concurrent changes of this behavioral intervention over the somatosensory-motor cortex.

Study Design

Participants. Thirteen people with SD (8 female, 5 male; mean age ± SD: 58.6 ± 12.5 years) were recruited through the University of Minnesota Fairview Loin's Voice Clinic and consented prior to study begin (see Table 1). Patients receiving Botulinum toxin are tested toward the end of their injection cycle when they were most symptomatic.

Experimental procedure. The experiment takes place in an acoustically shielded room. Participants sat on a comfortable chair and will avoid extra movements. Two small, encapsulated vibro-motors were attached bilaterally over the skin of the participant's laryngeal area (the voice box). Before the experiment, the severity of SD symptoms was assessed evaluated by (1) reading a series of sentences devised for speech examination in spasmodic dysphonia [1]; and (2) pronouncing the vowel /a/ three times, each lasting four seconds. The experimental procedure comprised: (1) laryngeal vibration, and (2) vowel vocalization accompanied by laryngeal VTS. During the vibration-only condition, laryngeal vibrators were alternately turned on (for 3 seconds) and off (for 3 seconds) for 50 repetitions, and then kept ON continuously for the final 3 minutes. During the second task, participants received a 250ms-long auditory cue and then vocalize the vowel /a/ continuously for 4 seconds. During the second half of this vocalization period of 4 seconds, laryngeal VTS was applied in two sets, each lasting about 17 minutes. Voice/speech quality was evaluated before the onset of the experiment, at the end of the first set, at the end of the second set, and 20 minutes after the cessation of VTS (retention).

Methodology

Measures of speech quality. Two voice measures were derived: (1) the number of voice breaks, and (2) the change in the cepstral peak prominence (CPP) of voice [21]. CPP is a measure of speech quality defined as the difference in amplitude between the cepstral peak and the corresponding value on the regression line that is directly below the peak. The higher the relative amplitude of the cepstral peak of a voice signal, the well-defined harmonic structure of the voice exists. For the analysis, speech signals were broken into 'voiced' and 'voiceless' segments and CPPS values were derived only for the 'voiced' periods. The PRAAT software [22] was used for the acoustic analysis of voice data. A certified speech-language pathologist identifies the number voice breaks per participant.

EEG signal processing and electrocortical measures. The EEGLab toolbox of MATLAB (The MathWorks, Natick, MA) was used for the offline analysis of all EEG data [23]. We obtained the event-related spectral perturbation (ERSP) of somatosensory and motor cortical electrodes in response to VTS, which is the logarithm of the mean event-related deviation in spectral power relative to the resting state at each frequency bin [24]. Band-specific measures will be computed for theta (4-8Hz), alpha (8-13Hz), beta (13-30Hz), and low gamma (30-49Hz). ERSP was extracted for 6 sites: CP5, C5, FC5, CP6, C6, and FC6. In addition, we obtained the event-related coherence (ERCOH) between pairs of somatosensory-motor cortical electrodes to evaluate the amount of synchronous activity between two different cortical sites [25]. ERCOH was derived for CP5-FC5 and CP6-FC6 electrode pairs.

Safety considerations. The study treatment was non-invasive. The application of VTS was classified as a non-significant risk by the Institutional review Board of the University of Minnesota.

Follow-up. This feasibility study was completed in one experimental session. Retention of the effect of VTS on markers of voice quality was assessed 20 minutes past posttest. No other follow-up visits associated with the research were required.

Data Management and Statistical Analysis

The Kolmogorov-Smirnov test was used for the assessment of the normality of the data. A non-parametric Wilcoxon signed-rank tests was used for statistical group comparisons. P-values were adjusted for multiple comparisons using false discovery rate correction based on the Benjamini-

Hochberg method [26]. The initial significance level was set to p-value = 0.05. Effect size is computed using Cohen's d.

Quality Assurance

Only the principal investigator and authorized laboratory staff have access and can manage the study data. Participant identifiable information is kept in a spreadsheet file along with a code for that participant (e.g. Subject #1 = S01) for the duration of the study. The identifier file and all other digital data are stored on a secure, password protected HIPAA compliant server. The other digital data will be files containing time-series data of EEG brain signals as well as data of the recorded voice signlas. Consent forms containing names and signatures of participants are kept in a locked file cabinet within a locked storage room. This storage room is within the laboratory, which is electronically locked 24 hours a day and only accessible for authorized personnel. Identifiable data will only be maintained for the duration of the study. No HIPAA data will not be shared.

Expected Outcomes of the Study

We expected that laryngeal VTS induces an alleviation of dystonia symptoms, i.e., an improvement of speech quality in at least in a subset of SD participants. We further expected that those participants with more severe voice symptoms showed a stronger response to laryngeal VTS.

Dissemination of Results and Publication Policy

In any publications or presentations, we will not include information that will make it possible to identify the study participants. However, records of the study may be reviewed by departments at the University of Minnesota with appropriate regulatory oversight.

Problems Anticipated

The study requires the application of laryngeal VTS via small, encapsulated vibratory motors, and the external recording of brain signals via EEG, both of which are completely non-invasive. For all study participants, there is a risk of feeling tired or fatigued from participating, because the study requires repeated voice articulations over a prolonged period (approx. 40 minutes). There is no other risk associated with the study.

Project Management

JK, JA, PW designed the experiment. SK, I-LY, AM collected the data. SK, I-LY, JA, AM, PW, and YZ, analyzed the data. GG served as clinical liaison. SK and JK wrote the manuscript with input from PW, YZ, and GG. JK supervised the work.

Ethics

The experimental procedure was approved by the University of Minnesota Institutional Review Board. Any publication or presentation related to this study will not include information that makes it possible to identify the study participants. However, the records of the study may be reviewed by departments at the University with appropriate regulatory oversight.

Informed Consent Forms

A copy of the approved informed consent form is available.

References

1 Woodson, G. E. in *Laryngeal Evaluation: Indirect Laryngoscopy to High-Speed Digital Imaging* (ed Katherine A. Kendall; Rebecca J. Leonard) Ch. 25, (Thieme, 2010).
2 Castelon Konkiewitz, E. *et al.* Service-based survey of dystonia in Munich. *Neuroepidemiology* **21**, 202-206 (2002).
3 Ludlow, C. L. Spasmodic dysphonia: a laryngeal control disorder specific to speech. *J Neurosci* **31**, 793-393 (2011).
4 Watts, C., Whurr, R. & Nye, C. Botulinum toxin injections for the treatment of spasmodic dysphonia. *Cochrance Database Sys Rev*, doi:10.1002/14651858.CD004327.pub2 (2004).
5 Simonyan, K., Berman, B. D., Herscovitch, P. & Hallett, M. Abnormal striatal dopaminergic neurotransmission during rest and task production in spasmodic dysphonia. *J Neurosci* **33**, 14705-14714, doi:10.1523/JNEUROSCI.0407-13.2013 (2013).
6 Ludlow, C. L., Yamashita, T., Schulz, G. M. & Deleyiannis, F. W. B. Abnormalities in long latency responses to superior laryngeal nerve stimulation in adductor spasmodic dysphonia. *Ann Otol Rhinol Laryngol* **104**, 928-935, doi:10.1177/000348949510401203 (1995).
7 Samargia, S., Schmidt, R. & Kimberley, T. J. Shortened cortical silent period in adductor spasmodic dysphonia: Evidence for widespread cortical excitability. *Neurosci Lett* **560**, 12-15, doi:https://doi.org/10.1016/j.neulet.2013.12.007 (2014).
8 Simonyan, K. *et al.* Focal white matter changes in spasmodic dysphonia: a combined diffusion tensor imaging and neuropathological study. *Brain* **131**, 447-459, doi:10.1093/brain/awm303 (2008).
9 Simonyan, K. & Ludlow, C. L. Abnormal activation of the primary somatosensory cortex in spasmodic dysphonia: an fMRI study. *Cereb Cortex* **20**, 2749-2759, doi:10.1093/cercor/bhq023 (2010).

10 Maschke, M., Gomez, C. M., Tuite, P. J. & Konczak, J. Dysfunction of the basal ganglia, but not the cerebellum, impairs kinaesthesia. *Brain* **126**, 2312-2322, doi:10.1093/brain/awg230 (2003).

11 Putzki, N. *et al.* Kinesthesia is impaired in focal dystonia. *Mov Dis* **21**, 754-760, doi:10.1002/mds.20799 (2006).

12 Patel, N., Hanfelt, J., Marsh, L. & Jankovic, J. Alleviating manoeuvres (sensory tricks) in cervical dystonia. *J Neurol Neurosurg Psychiatry* **85**, 882-884, doi:10.1136/jnnp-2013-307316 (2014).

13 Konczak, J., Aman, J. E., Chen, Y.-W., Li, K.-y. & Watson, P. J. Impaired limb proprioception in adults with spasmodic dysphonia. *J Voice* **29**, 777.e717-777.e723, doi:10.1016/j.jvoice.2014.12.010 (2015).

14 Kägi, G. *et al.* Sensory tricks in primary cervical dystonia depend on visuotactile temporal discrimination. *Mov Dis* **28**, 356-361, doi:10.1002/mds.25305 (2013).

15 Poisson, A. *et al.* History of the 'geste antagoniste' sign in cervical dystonia. *J Neurol* **259**, 1580-1584, doi:10.1007/s00415-011-6380-7 (2012).

16 Karnath, H., Konczak, J. & Dichgans, J. Effect of prolonged neck muscle vibration on lateral head tilt in severe spasmodic torticollis. *J Neurol Neurosurg Psychiatry* **69**, 658-660, doi:10.1136/jnnp.69.5.658 (2000).

17 Bianconi, R. & Van Der Meulen, J. P. The response to vibraion of the end organs of mammalian muscle spindels. *J Neurophysiol* **26**, 177-190, doi:10.1152/jn.1963.26.1.177 (1963).

18 Brown MC, E. I., Matthews PB. The use of vibration as a selective repetitive stimulus for Ia afferent fibres. *J Physiol* **191**, 31P-32P (1967).

19 Cordo, P., Gurfinkel, V. S., Bevan, L. & Kerr, G. K. Proprioceptive consequences of tendon vibration during movement. *J Neurophysiol* **74**, 1675-1688, doi:10.1152/jn.1995.74.4.1675 (1995).

20 Cordo, P. J., Gurfinkel, V. S., Brumagne, S. & Flores-Vieira, C. Effect of slow, small movement on the vibration-evoked kinesthetic illusion. *Exp Brain Res* **167**, 324-334, doi:10.1007/s00221-005-0034-x (2005).

21 Fraile, R. & Godino-Llorente, J. I. Cepstral peak prominence: a comprehensive analysis. *Biomed Signal Process Control* **14**, 42-54, doi:10.1016/j.bspc.2014.07.001 (2014).

22 Boersma, P. & van Heuven, V. Speak and unspeak with PRAAT. *Glot International* **5**, 341-347 (2001).

23 Delorme, A. & Makeig, S. EEGLAB: an open source toolbox for analysis of single-trial EEG dynamics including independent component analysis. *J Neurosci Method* **134**, 9-21, doi:10.1016/j.jneumeth.2003.10.009 (2004).

24 Makeig, S. Auditory event-related dynamics of the EEG spectrum and effects of exposure to tones. *Electroencephalog Clin Neurophysiol* **86**, 283-293, doi:10.1016/0013-4694(93)90110-H (1993).

25 Pfurtscheller, G. & Andrew, C. Event-related changes of band power and coherence: methodology and interpretation. *J Clin Neurophysiol* **16** (1999).

26 Benjamini, Y. & Hochberg, Y. Controlling the false discovery rate: a practical and powerful approach to multiple testing. *J Royal Stat Soc Series B* **57**, 289-300 (1995).
